# Self‐care status in patients with heart failure: Systematic review and meta‐analysis

**DOI:** 10.1002/nop2.805

**Published:** 2021-02-23

**Authors:** Ali Aghajanloo, Reza Negarandeh, Leila Janani, Kiarash Tanha, Sara‐Sadat Hoseini‐Esfidarjani

**Affiliations:** ^1^ Medical Surgical Nursing Department Faculty of Nursing and Midwifery Zanjan University of Medical Sciences Zanjan Iran; ^2^ Nursing and Midwifery Care Research Center School of Nursing and Midwifery Tehran University of Medical Sciences Tehran Iran; ^3^ Department of Biostatistics School of Public Health Iran University of Medical Sciences Tehran Iran

**Keywords:** heart failure, meta‐analysis, self‐care, systematic review

## Abstract

**Aim:**

To systematically review the status of self‐care in patients with heart failure through the Self‐Care of Heart Failure Index scale.

**Design:**

A systematic review and meta‐analysis.

**Methods:**

Following national and international databases were searched to retrieve eligible studies: PubMed, Web of Science, Embase, Google Scholar, Scientific Information Database and Magiran. The studies were screened and selected by two researchers. Data analysed through the random‐effects model, and the *I* ^2^ index was used to assess heterogeneity. Stata software version 12.0 was used for analysis. The PRISMA statement was used to report systematic review and meta‐analysis.

**Results:**

Of the 5,953 articles initially identified, 39 studies were included. The average score was estimated at 58.16 (CI: 54.39–61.94) for self‐care maintenance, 53.11 (CI: 49.17–57.05) for self‐care management and 58.66 (CI: 54.32–63.00) for self‐care confidence. Despite the high heterogeneity of the studies, the results indicated that self‐care practice is inadequate in all the three dimensions of self‐care (maintenance, management and confidence).

## 1 INTRODUCTION

Heart failure (HF) is an important public health problem; it is also a prevalent and complex syndrome (Roger, 2013). HF is a progressive and unpredictable heart disease that is caused by structural and functional disorders of the heart and is characterized by a range of symptoms (increased jugular venous pressure) and annoying signs (including dyspnoea and fatigue) (Kurmani & Squire, 2017). Heart failure is known as a pandemic syndrome that has affected a large number of people in the world. HF has affected approximately 15 million people in Europe, 6.5 million in the United States, and more than 37.7 million worldwide (Benjamin et al., ,^2018^, ^2019^; Ziaeian & Fonarow, 2016). Improving the survival rate in patients with myocardial infarction and high blood pressure has increased the number of patients with heart failure (Liu et al., 2014). The prevalence of HF in adults ranges from 1%–2%, and it is more than 12% in adults over 80 years of age (Vellone et al., 2017). Therefore, HF is more prevalent in countries with a large elderly population.

Despite the advances in treatment, heart failure is still associated with high levels of hospitalization, low quality of life, increased demands for healthcare services, and premature death (Hjelm et al., 2015). HF is the leading cause of hospitalization and re‐hospitalization in the elderly (Lee et al., 2018). Frequent hospitalization in patients with heart failure is considered a burden on healthcare systems and negatively affects patients’ long‐term outcomes (Zeng et al., 2017). The prognosis of patients with heart failure is very poor, and their life expectancy is often fewer than 5 years (Liu et al., 2014). Therefore, the mortality rate of the patients with heart failure is high, that is, heart failure is considered the cause of one out of each nine deaths (Benjamin et al., 2019). Besides, heart failure affects the quality of life of the patients (QOL). Studies have shown that the quality of life among patients with HF is worse than that of other chronic diseases due to severe symptoms, frequent hospitalizations and use of emergency services (Moradi et al., 2020; Vellone et al., 2017). Given the chronic nature of HF disease and factors such as decreased physical ability, impaired social and personal relationships, and inability to perform work‐related duties, patients also face financial problems and increased medical costs (Sahebi et al., 2015).

Since HF is a progressive and debilitating disease, it requires lifelong management of the disease to achieve the desired therapeutic results (Lee et al., 2017). The goals of disease management for people with heart failure are to improve self‐care, minimize adverse effects, reduce hospitalization and improve quality of life (Liu et al., 2014). Treatment guidelines recommend both pharmacological and non‐pharmacological methods for disease management. Self‐care is a crucial non‐pharmacological strategy in preventing recurrent hospitalizations and improving health outcomes (Cao et al., 2016). In other words, self‐care is the foundation and an essential part of the management of heart failure (Tung et al., 2012). Researchers have found that self‐care can improve the health status of patients with HF (Tung et al., 2012).

Moreover, if patients adhere to constant self‐care, they can have better control over their HF. Promoting self‐care behaviours in patients helps them to be able to perform daily activities more efficiently and also to improve their quality of life by managing their social behaviour (Sahebi et al., 2015). Adequate self‐care can help prevent aggravating the heart failure situation and improve clinical outcomes (Jonkman et al., 2016). Numerous studies have shown that there is a positive relationship between self‐care and improving physical performance, survival and quality of life, as well as reducing hospitalization, healthcare costs and mortality rate in patients with HF (Clark et al., 2015; Hamar et al., 2015; Uchmanowicz et al., 2017).

Self‐care is referred to as a process of individuals’ participation in taking responsibility for managing various aspects of their health and adopting some behaviours to prevent disease, limiting illness and restore health (Cameron, Worrall‐Carter, Page, Riegel, et al., 2010). Exhibiting self‐care behaviours can help increase longevity, maintain functional capacity, maintain independence and quality of life, reduce hospitalization and reduce pain as well as personal costs (Bell et al., 2016).

Self‐care includes maintenance, management and confidence. Self‐care maintenance reflects the behaviours that are exhibited to maintain physiological and emotional stability (Cameron et al., 2009). Self‐care maintenance includes daily weighing, adherence to a low‐sodium diet, regular exercise, strict adherence to medication, immunization and regular periodic visits (Dickson et al., 2013). Self‐care management refers to making the right decisions on the control of the symptoms when they occur. Self‐care management is an active and deliberate process that is necessary and important in the management of heart failure (Cameron et al., 2009). Self‐care confidence or self‐efficacy refers to patients' perceived ability to participate in each stage of the self‐care process. Self‐efficacy is a crucial part of the self‐care process (Buck et al., 2015) so that self‐care confidence can have a positive effect on self‐care maintenance and self‐care management (Ausili et al., 2016; Dickson et al., 2013).

Healthcare providers need to understand the patient's self‐care status in order to provide appropriate services for these patients. Therefore, several studies have been conducted on the level of self‐care behaviours among patients with HF around the world. Numerous scales have been used in these studies to measure the type and extent of self‐care behaviours in patients with HF. However, most of these studies have used one version of the Self‐Care of Heart Failure Index (SCHFI). The SCHFI has three dimensions that measure different aspects of self‐care (self‐care maintenance, self‐care management and self‐care confidence), and then the scores are reported as a standardized score ranging from 0–100. It is crucial to provide a clear description of patients' self‐care status by pooling the results of primary studies for conducting further research and designing effective interventions. The literature review revealed that, while there are some systematic reviews about self‐care among people with heart failure (Clark et al., 2014; Harkness et al., 2015; Zhao et al., 2020), no current systematic reviews describe HF patients' self‐care practices. Therefore, the present study was conducted to assess self‐care status in patients with HF through the SCHFI scale.

## 2 METHOD

This study is a systematic review and meta‐analysis on the self‐care status in patients with heart failure. The results of the present study would be reported based on the Preferred Reporting Items for Systematic Reviews and Meta‐Analysis (PRISMA) Statement (Moher et al., 2009) (See Appendix S1). The study protocol was registered in the PROSPERO (International prospective register of systematic reviews) database (no. CRD42018090796).

### 2.1 Search strategy

A systematic electronic search was conducted through PubMed, Web of Science and Embase between 2004 (Since SCHFI first publication) and 2018 to find the related studies. A comprehensive search strategy was used to increase the search sensitivity and access to the most relevant articles. For this purpose, the researchers selected these keywords: "heart failure," "congestive heart failure," "self‐care" or "self‐management" and also similar spellings of them. Complementary searches were also performed on Google Scholar, SID (Scientific Information Database) and Magiran, as well as manual searches through existing journals and related articles. The sample search on PubMed reads as follows:

*(Heart Diseases[Title/Abstract] OR heart failure[Title/Abstract] OR congestive heart failure[Title/Abstract] OR Heart Failure, Diastolic[Title/Abstract] OR Heart Failure, Systolic[Title/Abstract] OR cardiac failure[Title/Abstract]) AND (Self‐Care[Title/Abstract] OR Self‐Management[Title/Abstract] OR disease management[Title/Abstract] OR self‐administration[Title/Abstract] OR self‐medication[Title/Abstract] OR self‐monitoring[Title/Abstract])*.

### 2.2 Eligibility criteria

The observational studies (cross‐sectional and cohort studies) to assess patients’ self‐care status among individuals over 18 years old with HF would primarily be included in the review. These studies were reported to have implemented one of the 22‐item, 19‐item, 17‐item and 15‐item versions of the SCHFI scale to measure self‐care behaviours. The articles were published between 2004–2018. The studies were published in English and Persian and were available in full text. The exclusion criteria include interventional studies, clinical trials, study protocols, review studies, letters to the editors, abstracts, instrumental psychometrics, experimental studies or qualitative studies. If there were several similar articles on the same subject or secondary analysis of the data, the study that has provided more complete results would be included in the study.

### 2.3 Study selection

All the reviewed articles were entered into endnote software, and duplicated items were deleted. The remaining articles were then reviewed and screened by two authors in terms of the title and abstract. If there were a disagreement, the full text of the article would be read. In the next step, the full texts of all the selected articles were reviewed separately by two authors in terms of entry criteria, and then some articles would be selected for the final review.

### 2.4 Risk of bias assessment

Two authors independently conducted the quality appraisal of the articles using the modified Newcastle‐Ottawa Scale adapted for cross‐sectional studies. Each included study was assessed in "star system" from three broad perspectives: (a) selection (Representative of a sample, Sample size, Non‐respondents), (b) comparison (Adjusted or unadjusted for variables) and (c) outcome (Assessment method and Statistical analysis). The selection, comparison and outcome would receive a maximum of three stars, two stars and three stars, respectively, which becomes eight stars in total (Wells et al.,) (See Appendix S2). The higher number of stars indicates the low risk of bias in the study.

### 2.5 Data extraction

Two independent investigators extract data using the data collection form, which has been developed and piloted for assessing its usability. The following data were extracted from the study reports: first author name, year of publication, country, type of study, sample size, sampling technique, the setting of the study, participant profile (age, sex, New York Heart Association Classification and ejection fraction), used instrument, analysis, the mean and standard deviation of self‐care in the three dimensions of self‐care (maintenance, management and confidence). For selected studies, data were extracted independently by two researchers. During the review process, any disagreement between the two authors would be resolved through discussion or referral to a third researcher.

### 2.6 Statistical analysis

The data were analysed using STATA software, version 12 (STATA Corporation). We pooled the data, which were weighted by the inverse variance of the individual studies, to derive an overall mean and the associated 95% confidence interval (CI). Heterogeneity was evaluated to determine the extent of variation in effect estimates due to heterogeneity rather than chance. Heterogeneity among the primary studies was assessed by the forest plots, *χ* ^2^ test (with significance defined at the a‐level of 10%) and *I* ^2^ statistic. A random‐effects model was used because of high heterogeneity (*I* ^2^ > 50%); any value lower than .05 (*p* < .05) was considered statistically significant. The characteristics of the included studies were descriptively summarized using a structured table.

## 3 RESULTS

**FIGURE 1:**
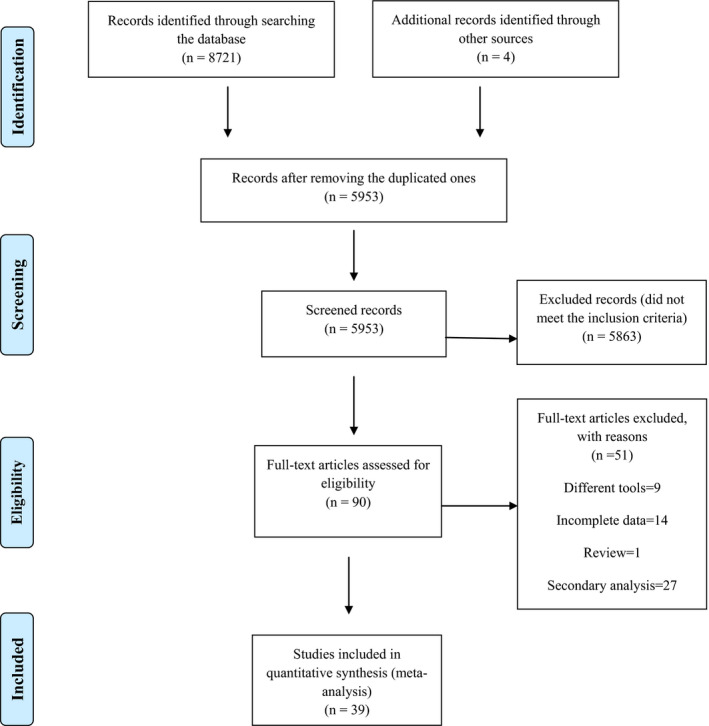
Flow diagram of the study selection process based on PRISMA guidelines

Searches in predetermined electronic databases found 8,721 articles, an additional four documents found with hand searching. After deletions of the duplicated ones, 5,953 articles were reviewed in terms of title and abstract. Five thousand eight hundred and sixty‐three articles were irrelevant and did not meet the inclusion criteria, leaving 90 for full‐text reviewing. After reviewing the full texts, 51 articles that did not meet the eligibility criteria for the present study were excluded in the next step. In the end, 39 studies were approved and included in the review process and the meta‐analysis phase. The diagram (PRISMA flow diagram) shows the inclusion procedure for the studies (Figure 1).

The selected articles included 36 cross‐sectional studies, two prospective studies and one cohort study. Those studies were conducted in Asia (Iran, China, Korea, Taiwan, Thailand, and Jordan), North America (USA, Mexico, and Canada), Europe (Italy and UK), Brazil and Australia. There were 20 hospital studies, 15 outpatient studies and four studies based on the combination of outpatient and hospital studies. Different versions of the SCHFI tool, including SCHFI 22, SCHFI 19, SCHFI 17 and SCHFI 15, have been used in these studies. All scores in the studies were standardized in a range of 0–100, and any score above 70 was considered good self‐care (Riegel et al., 2009).

Regarding the quality of articles, the minimum and maximum stars received were 2 and 7, respectively, with a median of 3 and an interquartile range of 2 (See Appendix S2).

The total sample size included 8,958 patients (New York Heart Association Functional Classification: I–IV). The samples included 5,428 males and 3,530 females. The sample size of each study varied from 17–2,082 individuals, with an average of 230 samples per study. The mean age range of the patients was 55–80 years. The average ejection fraction was reported in 20 studies with a range of 21%–54% on average.

The average score for self‐care maintenance was reported in 36 studies, ranging from 20 (Zamanzadeh et al., 2012)–76 (Chen et al., 2011). The average score for self‐care maintenance was above 70 in just four studies, and it was reported below 70 in 32 other studies.

The average score for self‐care management was reported in 35 studies, ranging from 14 (Zamanzadeh et al., 2012)–68 (Davis et al., 2015). The average score for self‐care management was reported below 70 in all the studies.

The average score for self‐care confidence was reported in 37 studies, ranging from 13 (Zamanzadeh et al., 2012)–85 (Tung et al., 2012). The average score for self‐care confidence was above 70 in just three studies, and it was reported below 70 in 34 studies. The full characteristics of the included studies are listed in Table 1.

**TABLE 1:** The characteristics of selected articles

Author/year	Study design				Participants				Instrument	Self‐care		
	Type of study	Country	Sample size	Setting	Age, years (mean ± *SD*)	Sex Male %	NYHA	LVEF, % (mean ± *SD*)		self‐care maintenance, *M*(SD)	self‐care Management, *M*(SD)	self‐care confidence, *M*(SD)
Zou et al. (2017)	Cross‐sectional	China	321	Hospital	63.6 ± 10.6	51.4	II–IV	(>40%) = 83.8 (<40%) = 16.2	SCHFI 22 Items	48.4 (15.9)	54.3 (19.3)	57.1 (13.4)
Zamanzadeh et al. (2012)	Descriptive	Iran	80	Hospital	63.5 ± 11.2	46.3	III–IV	24.8 ± 9.2	SCHFI 22 Items	20.2 (13.3)	14.3 (14.5)	13.6 (14.0)
Wu et al. (2017)	Prospective, observational	The United States of America (USA)	173	Hospital	61 ± 12	66	I–IV	(<40%) = 63% (>41%) = 37%	SCHFI 19 items	62.1 (17.5)	60.4 (21.9)	67.8 (16.2)
Vellone et al. (2016)	Descriptive	USA	280	outpatient	62 ± 12.5	64.3	I–IV		SCHFI 22 Items	66.8 (11.9)	67.4 (18.7)	75.8 (14.1)
Vellone et al., 2014)	Cross‐sectional	Italy	138	outpatient	73.6 ± 9.6	67.4	I–IV	43 ± 10.8	SCHFI 22 Items	53.7 (15.5)	51.0 (18.5)	49.8 (18.5)
Tung et al. (2014)	Descriptive cross‐sectional	Taiwan	98	Hospital	67.36 ± 14.78	75.5	II–III		SCHFI 22 Items	66.01 (17.98)	63.06 (22.90)	74.21 (23.74)
Tung et al. (2012)	Descriptive, cross‐sectional, correlational	Taiwan	86	Hospital	65.73 ± 12.56	73	II–III	36 ± 3.96	SCHFI 22 Items	53.95 (19.10)	53.37 (25.81)	85.87 (21.80)
Tsai et al. (2015)	Cross‐sectional correlational	Taiwan	71	Hospital	<65 = 32.4 >65 = 67.6	38	II–III		SCHFI 22 Items	47.93 (18.23)	29.73 (26.14)	40.02 (26.94)
Trivedi et al. (2012)	Cross‐sectional	England	23	Outpatient		100%	I–III		SCHFI	59.7 (17.3)	54 (19.4)	53.3 (28.2)
Tawalbeh et al. (2017)	Cross‐sectional descriptive	Jordan	226	Hospital	56.92 ± 12.29	61.9			SCHFI 22 Items	53.89 (29.77)	57.56 (29.16)	45.07 (35.67)
Siabani et al. (2016)	Cross‐sectional	Iran	231	Hospital	66 ± 13	51.5	I–III		SCHFI 22 Items	33.8 (10.65)	32.2 (12.04)	43.6 (15.60)
Schnell‐Hoehn et al. (2009)	Cross‐sectional	Canada	65	Outpatient clinic	59 ± 13	33	I–IV	(≤20%) = 34% (21%–40%) = 55% (≥41) = 11%	SCHFI 22 Items		43.9 (24.6)	63.9 (16)
Salyer et al. (2012)	Cross‐sectional	USA	97	Hospital	56.33 ± 13.73	56.7	I–IV	25.77 ± 8.3	SCHFI 15 Items	69.59 (15.56)	62.20 (19.81)	66.30 (17.01)
Sahebi et al. (2015)	Cross‐sectional	Iran	287	Hospital	60.2 ± 43.1	67.7	I–IV	21.01 ± 7.21	SCHFI	57.7 (15.11)	56.05 (21.35)	61.7 (21.52)
Riegel et al. (2011)	Cross‐sectional descriptive	USA	689	Hospital	61.3 ± 12.5	64.4	I–IV	30.4 ± 14.6	SCHFI 15 Items	57.2 (22.8)	57.8 (22.6)	58.4 (19.6)
Riegel, Driscoll, et al. (2009))	Descriptive, comparative	USA, Australia, Thailand, Mexico	2,082	Outpatient clinic & Hospital	66.56 ± 12.39	62.72	I–IV		SCHFI 15 Items	65.35 ± 8.24	56.48 (10.15)	66.93 (9.50)
Quinn et al. (2010)	Descriptive cross‐sectional, correlational	USA	70	home healthcare	(40–59) = 14.2% (60–79) = 68.5% (80–89) = 17.1%	40	II–III		SCHFI 15 Items		57.9 (15.8)	60.5 (19.0)
Lyons et al. (2017)	Cross‐sectional	USA	60	Outpatient clinic	59.45 ± 11.92	66.7	I–IV		SCHFI	71.53 (13.93)	65.52 (21.83)	63.35 (19.25)
Levin et al. (2014)	Cohort	USA	17	Outpatient	80.2 ± 5.1	41.2	II–III	41 ± 14	SCHFI 15 Items	73.1 (10.9)	58.0 (20.6)	62.1 (21.9)
Lee, et al. (2017)	Cross‐sectional	USA	206	Outpatient clinic	60 ± 11.6	66.5	I–IV		SCHFI 17 Items	49.6(22.5)	54.7(21.3)	56.5(22.0)
Lee et al. (2015)	Cross‐sectional, observational	USA	311	Outpatient clinic	60 ± 11.9	64.6	I–IV	(<40%) = 64.3% (>40%) = 35.7%	SCHFI‐Management subscale		55.5 (20.5)	
Lee et al. (2012)	Cross‐sectional	USA and Australia	207	Outpatient and inpatient	72.9 ± 6.3	41.5	III–IV	31.7 ± 15.5	SCHFI‐17	54.1 (23)	56 (22.9)	55.7 (19.6)
Cameron et al. (2010)	Cross‐sectional	Australia	143	Hospital	72 ± 11	73	I–IV		SCHFI‐15	68 (15)	54 (19)	65 (17)
Cao et al. (2016)	Cross‐sectional	China	127	Hospital	64.9 ± 12.34	61.4	II–IV		SCHFI‐22	39.71 (13.17)	35.98 (15.35)	49.69 (18.42)
Cené et al. (2013)	Cross‐sectional	USA	150	outpatient clinic	61 ± 12	49	I–IV	(≥55) = 39% (40%–55%) = 24% (<40%) = 37%	SCHFI‐22	70 (14)	57 (24)	65 (17)
Vellone et al. (2014)	Descriptive comparative	USA	121	Hospital	71.24 ± 9.8	49.6			SCHFI‐22	63.22 (18.37)	57.18 (25.05)	66.02 (22.40)
Chang et al. (2017)	Cross‐sectional	Taiwan	201	outpatient clinics	62.4 ± 11.4	69.7	I–III		SCHFI	58.65 (17.20)		62.40 (22.75)
Chen et al. (2011)	Cross‐sectional	USA	49	Hospital & clinics	72 ± 13.3	32.7			SCHFI‐15	76.74 (20.98)	65.05 (24.47)	68.60 (22.31)
Chriss et al. (2004)	Descriptive‐correlational	USA	66	Hospital	71 ± 13.3	43.9	I–IV	(≥50) = 37.9% (41%–49%) = 6.9% (<40%) = 55.2%	SCHFI‐15	64.8 (18.6)		
Cocchieri et al. (2015)	Cross‐sectional	Italy	1,192	Cardiovascular centres	72.36 ± 11.2	58.2	I–IV	44.6 ± 10.9	SCHFI‐22	55.26	53.18	54.57
Conceicao et al. (2015)	Descriptive cross‐sectional	Brazil	116	ambulatory care	57.7 ± 11.3	54.3	I–III	40.4 ± 12.9	SCHFI‐22	50.5 (15.7)	50 (20.3)	58.1 (18.2)
Davis et al. (2015)	Descriptive‐correlational	USA	125	hospital	59 ± 13	53	I–IV		SCHFI‐22	63.57 (19.12)	68.35 (20.24)	64.99 (16.06)
Dennison et al. (2011)	Descriptive comparative	USA	95	hospital	59 ± 14	51		(≥40%) = 45% (<40%) = 55%	SCHFI‐22	56.82 (17.12)	63.64 (18.29)	65.02 (16.34)
Harkness et al. (2014)	Observational, cross‐sectional	Canada	100	outpatient clinics	72.4 ± 9.8	68	I–III		SCHFI‐22	67.1 (16)	51.1 (23.6)	55.4 (20)
Heo et al. (2008)	Cross‐sectional, correlational	USA	122	outpatient clinics, hospital	60.3 ± 12	63	I–IV		SCHFI	62.3 (19)		69.2 (17.8)
Hooker et al. (2018)	Cross‐sectional	USA	99	hospital	65.6 ± 12.4	79	I–IV	(≥50%) = 38.6% (40%–49%) = 20.5% (30%–39%) = 13.6% (<30%) = 27.3	SCHFI	66.8 (15)		63.3 (21.3)
Kim et al. (2015)	Prospective study	Korea	86	outpatient clinic	58.3 ± 12.9	67.4	I–IV	51.2 ± 14.6	SCHFI‐22	55.4 (14.3)	34 (12.8)	52.1 (17.6)
Farghadani et al. (2018)	Cross‐sectional	Iran	100	hospital	55.13 ± 13.76	59	I–IV		SCHFI‐22	56.59 (12.97)	53.94 (15.41)	58.82 (17.47)
Mansouri et al. (2018)	Cross‐sectional	Iran	248	Hospital	62.54 ± 11.35	61.3	I–III	30.77 ± 9.07	SCHFI‐22	52.85 (12.21)	55.27 (14.44)	26.57 (20.78)

Abbreviations: LVEF, Left ventricular ejection fraction; NYHA, New York Heart Association Classification.

**FIGURE 2:**
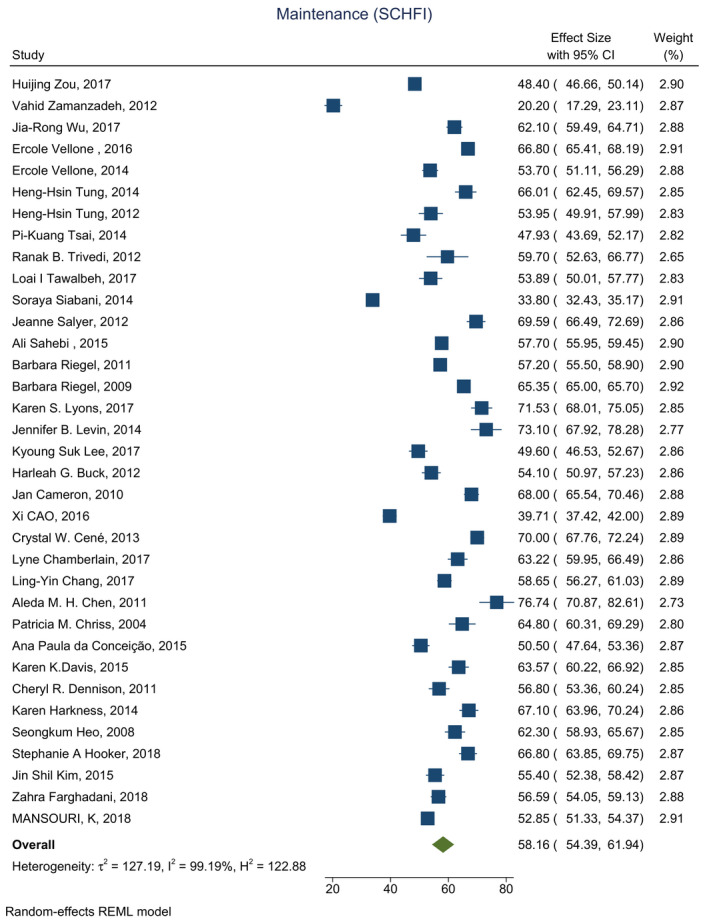
Forest plot of self‐care maintenance in HF patients

**FIGURE 3:**
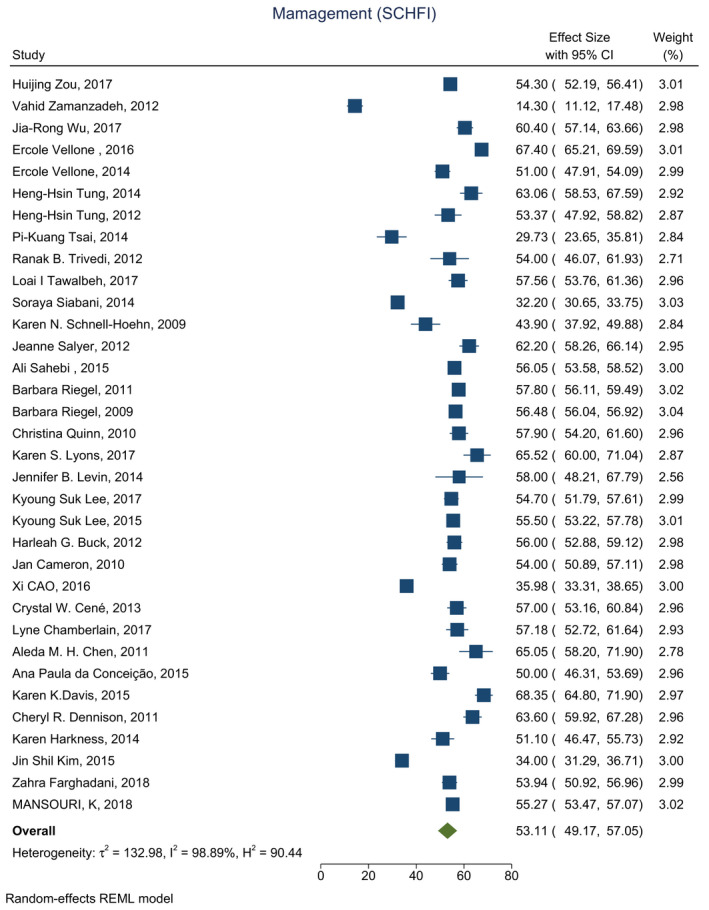
Forest plot of self‐care management in HF patients

**FIGURE 4:**
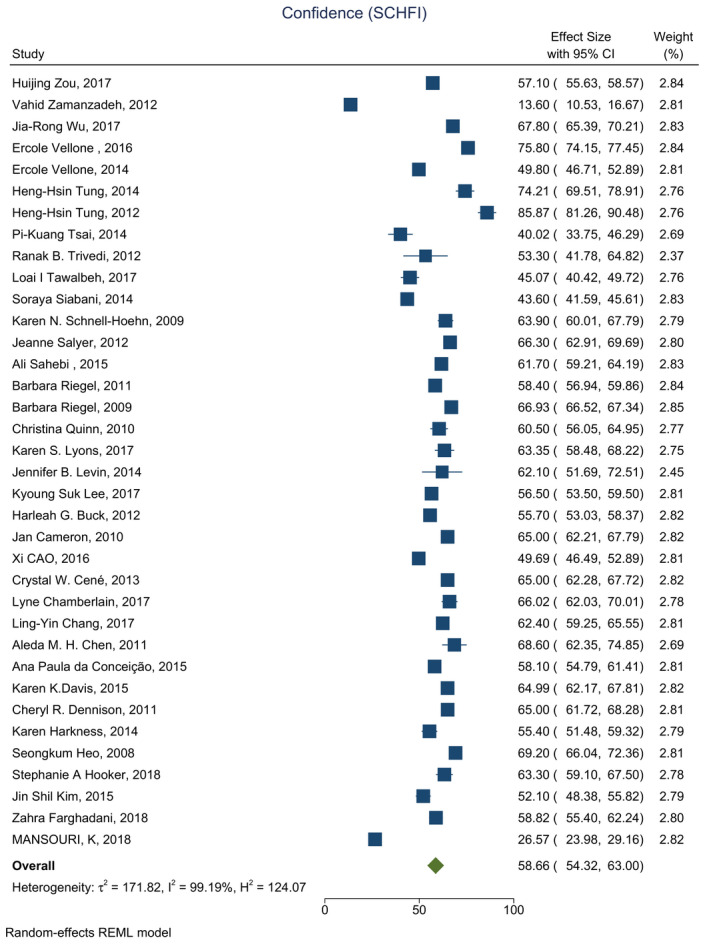
Forest plot of self‐care confidence in HF patients

Based on the results of the meta‐analysis, the average score was estimated 58.16 (CI: 54.39–61.94) for self‐care maintenance, 53.11 (CI: 49.17–57.05) for self‐care management and 58.66 (CI: 54.32–63.00) for self‐care confidence (Figures 2, 3, 4).

Due to the high level of heterogeneity, the meta‐regression analysis was used to find the source of the heterogeneity. For this purpose, an analysis was performed based on the country of study, study design, clinical setting and different versions of SCHFI tools, but had no significant effect on reducing the heterogeneity of studies. Also, analysis by excluding studies with outliers findings no resolve heterogeneity. Therefore, the results should be interpreted with caution due to the high level of heterogeneity.

## 4 DISCUSSION

The purpose of this systematic review and meta‐analysis study was to determine the self‐care status in patients with HF using the SCHFI scale. The results showed that the pooled average self‐care maintenance, self‐care management and self‐care confidence were 51.92, 44.46 and 45.19, respectively. The results also indicated that the average self‐care in patients with HF is lower than the acceptable norm (70 or higher) in all the three dimensions of self‐care. Although the meta‐analysis results should be interpreted with caution due to the high heterogeneity of the studies, the findings of this study showed that patients with HF showed poor self‐care behaviours.

Self‐care is the foundation of heart failure treatment (Clark et al., 2014). Studies have shown that moderate to high self‐care levels can affect outcomes and disease burden (Auld et al., 2018; Clark et al., 2014). However, self‐care is a complex process influenced by individual, contextual and situational factors (Harkness et al., 2015). Factors that affect self‐care include experience and skills, motivation, habits, cultural beliefs and values, functional and cognitive abilities, confidence, support and access to care (Jaarsma et al., 2017). Self‐care in patients with heart failure involves a wide range of lifestyle changes that may be affected by predisposing and/or enabling factors (Schnell‐Hoehn et al., 2009). However, it is difficult for patients to comply with all of them (Gallacher et al., 2011). As a result, patients are often inconsistent in adherence to self‐care. In fact, for most of the patients, taking medications, following a visit or contacting caregivers when necessary are much easier than exercising and reducing salt intake (Vellone et al., 2017).

Studies have shown that the self‐care process is not easily understood by most of the patients (Cameron et al., 2009). In other words, it is not easy for many patients with HF to learn self‐care skills (Cameron, Worrall‐Carter, Page, Riegel, et al., 2010). Cognitive and functional limitations, old age, low level of education, psychological problems, and insufficient social support are factors that can disrupt patients' self‐care (Santesmases‐Masana et al., 2019). Self‐care maintenance and management can prevent worsening and the need for complex and invasive HF treatments (Lee et al., 2009). But patients disability, disease burden and progression undermine adherence to recommended self‐care behaviours (Toukhsati et al., 2015). Health literacy is also associated with patients' ability to self‐care. Health literacy is health‐related knowledge, skills and experience that enables patients to recognize their health status and how they manage their healthcare (Santesmases‐Masana et al., 2019). Therefore, to improve patients 'self‐care knowledge and skills, patients' low health literacy should also be considered (Toukhsati et al., 2015).

Self‐care confidence affects the whole self‐care process. People with high self‐efficacy can frequently exhibit self‐care behaviours and are more likely to have better control over the disease's symptoms (Buck, 2009). Therefore, any attempt to improve self‐care confidence can directly affect self‐care maintenance and self‐care management (Buck, 2009). On the other hand, comorbidities reduce self‐efficacy, which, in turn, reduces self‐care behaviours (Buck et al., 2015). Those interventions designed to improve self‐care confidence can also improve self‐care even in patients with cognitive impairment (Vellone et al., 2015). The present study results indicated a low level of self‐care confidence among patients so that the average score of self‐care confidence was only acceptable in three studies (8% of the total studies). This can, to a large extent, explain the reasons for poor maintenance and management of self‐care among the patients in these studies.

It should be emphasized that self‐care can only work satisfactorily with the cooperation of the health system and professionals (Toukhsati et al., 2015). Therefore, having a systematic approach to comprehensive education and counselling with an emphasis on knowledge, skills and behaviours can enhance patients' self‐care. Also, adopting strategies such as early and targeted screening of patients to identify and address problems such as low health literacy, cognitive, physical and psychosocial disorders can be helpful (Toukhsati et al., 2015).

Self‐care is a multidimensional phenomenon, not a linear process. Besides, it is not affected only by some specific factors. The complexity of the symptoms and problems associated with HF increases the challenges of living with the disease (Cameron et al., 2009). Therefore, healthcare providers need to know that self‐care requires learning and gaining experience. They should take an individualized approach to develop HF patients' self‐care behaviours, emphasizing how and not just what. It is also helpful to create a safe environment for patients to discuss their efforts about how to self‐care. Also, the supportive role of healthcare providers is essential in improving patients' self‐care.

### 4.1 Limitations

Since significant heterogeneity was detected, regardless of using random‐effects meta‐analysis models, the result of this meta‐analysis should be interpreted with caution. Also, the moderate quality of some included studies can affect the precision of the estimation in this systematic review and meta‐analysis. Given that only Persian and English articles were included in the analysis, the results have limited generalizability due to the lack of articles from different languages.

## 5 CONCLUSION

Despite the high heterogeneity of the studies, the results indicated that self‐care practice is inadequate in all the three dimensions of self‐care (maintenance, management and confidence). Healthcare providers need to design specific and effective programs tailored to each patient's status and improve self‐care status among patients with heart failure.

## 6 ETHICS APPROVAL

The study protocol was registered in the PROSPERO (International prospective register of systematic reviews) database (no. CRD42018090796).

## CONFLICT OF INTEREST

The authors have declared that no competing interests exist.

## AUTHOR CONTRIBUTIONS

AA: design of study, literature search, data extraction and drafting of manuscript. RN: design of study, supervision of Review and Meta‐Analysis and revising of manuscript. LJ: design of study, statistical analysis and revising of manuscript. KT: statistical analysis and revising of manuscript. SSHE: literature search, data extraction and drafting of manuscript.

## CONSENT TO PARTICIPATE

This is a systematic review and consent to participate is Not applicable.

## CONSENT FOR PUBLICATION

Not applicable.

Manuscript did not include details, images or videos relating to an individual person.

## AVAILABILITY OF DATA AND MATERIAL

The datasets analysed during the current study are available from the corresponding author on reasonable request.

## AUTHORS AGREEMENT STATEMENT

All authors confirm that the manuscript has been read and approved by all named authors and that there are no other persons who satisfied the criteria for authorship but are not listed.

## Supporting information

Appendix S1

Appendix S2

## Data Availability

The data that support the findings of this study are available from the corresponding author upon reasonable request.
