# Supplementary material for: Self‐care status in patients with heart failure: Systematic review and meta‐analysis
Source: Nurs Open. 2021 Feb 23;8(5):2235–48. doi: 10.1002/nop2.805 (PMC8363344; doi:10.1002/nop2.805)
Supplement: Supplementary file 2 — Appendix S2 [file NOP2-8-2235-s001.docx]

**Appendix S2**

**Criteria for the Newcastle-Ottawa Scale regarding star allocation to assess the quality of studies**

| **Outcome (maximum** **3 Star)** | | **Comparability (maximum** **2 Star)** | **Selection (maximum** **3 Star )** | | | **criteria** |
| --- | --- | --- | --- | --- | --- | --- |
| Statistical test | Assessment of outcome |  | non-respondents | Sample size | Representative of sample |  |
| Reported  p-value | Self-report | Adjusted for one of these factors:  1-age  2-gender  3-NYHA classification  4-ejection fraction  5-comorbidity | <70% | > 300 | 1-all subject  2-random sampling  3-non random sampling(At least more than one setting) | One Star awarded |
| - | 1-independent blind assessment  2-record linkage | Adjusted for more than one of these factors:  1-age  2-gender  3-NYHA classification  4-ejection fraction  5-comorbidity | - | - | - | two Star awarded |
| No description | No description | Did not Adjusted for age, gender, NYHA classification, ejection fraction, and comorbidity | 1->70%  2- No description | 1- <300  2- No description | 1- non-random sampling(one setting-a group of users)  2- No description | Star not awarded |

**Quality assessment of studies using a modified Newcastle-Ottawa scale for assessing studies in the systematic review**

| **Total**  **(maximum** **8 Star)** | **Outcome^©^ (maximum** **3 Star)** | | **Comparability***  **(maximum** **2 Star)** | **Selection ^#^ (maximum** **3 Star )** | | | **First Author** |
| --- | --- | --- | --- | --- | --- | --- | --- |
|  | Statistical test | Assessment of outcome |  | non-respondents | Sample size | Representative of sample |  |
| 7 | * | * | ** | * | * | * | Zou et al. (2017)^40^ |
| 2 | - | * | - | * | - | - | Zamanzadeh et al. (2012)^23^ |
| 2 | * | * | - | - | - | - | Wu et al. (2017)^41^ |
| 4 | * | * | - | * | - | * | Vellone et al. (2016)^42^ |
| 4 | * | * | - | * | - | * | Vellone et al. (2014)^43^ |
| 3 | * | * | - | * | - | - | Tung et al. (2014)^44^ |
| 3 | * | * | - | - | - | * | Tung et al. (2012)^15^ |
| 3 | * | * | - | * | - | - | Tsai et al. (2015)^45^ |
| 2 | * | * | - | - | - | - | Trivedi et al (2012)^46^ |
| 6 | * | * | ** | * | - | * | Tawalbeh et al. (2017)^47^ |
| 3 | * | * | - | * | - | - | Siabani et al. (2016)^48^ |
| 2 | * | * | - | - | - | - | Schnell-Hoehn et al. (2009)^35^ |
| 5 | * | * | ** | * | - | - | Salyer et al. (2012)^49^ |
| 3 | * | * | - | * | - | - | Sahebi et al. (2015)^12^ |
| 5 | * | * | - | * | * | * | Riegel et al. (2011)^50^ |
| 6 | * | * | ** | - | * | * | Riegel et al. (2009)^51^ |
| 3 | * | * | - | - | - | * | Quinn et al. (2010)^52^ |
| 4 | * | * | ** | - | - | - | Lyons et al. (2017)^53^ |
| 2 | * | * | - | - | - | - | Levin et al. (2014)^54^ |
| 5 | * | * | * | * | - | * | Lee et al. (2017)^55^ |
| 7 | * | * | ** | * | * | * | Lee et al. (2015)^56^ |
| 4 | * | * | * | - | - | * | Buck et al.(2012)^57^ |
| 3 | * | * | * | - | - | - | Cameron et al.(2010)^39^ |
| 3 | * | * | - | - | - | * | Cao et al.(2016)^14^ |
| 3 | * | * | - | - | - | * | Cene et al.(2013) ^58^ |
| 4 | * | * | * | - | - | * | Chamberlain et al.(2017)(47) |
| 2 | * | * | - | - | - | - | Chang et al. (2017)^59^ |
| 2 | * | * | - | - | - | - | Chen et al.(2011)^24^ |
| 4 | * | * | * | - | - | * | Chriss et al.(2004)^60^ |
| 5 | * | * | * | - | * | * | Cocchieri et al.(2015)^61^ |
| 2 | * | * | - | - | - | - | da Conceicao et al.(2015)^62^ |
| 3 | * | * | * | - | - | - | Davis et al.(2015)^25^ |
| 4 | * | * | * | * | - | - | Dennison et al.(2011)^63^ |
| 5 | * | * | ** | - | - | * | Harkness et al.(2014)^64^ |
| 3 | * | * | * | - | - | - | Heo et al.(2008)^65^ |
| 2 | * | * | - | - | - | - | Hooker et al. (2018)^66^ |
| 3 | * | * | * | - | - | - | Kim et al.(2015)^67^ |
| 2 | * | * | - | - | - | - | Farghadani et al.(2018)^68^ |
| 3 | * | * | - | - | - | * | Mansouri et al.(2018)^69^ |
